# Social reintegration of cisgender and transgender women post-incarceration in Brazil: policies and challenges

**DOI:** 10.1186/s40352-024-00285-6

**Published:** 2024-07-11

**Authors:** Raquel B. Miranda, Alejandro Goldberg, Ximena Pamela Díaz Bermúdez

**Affiliations:** Post-Graduation Program of Collective Health, University of Brasilia, Campus Universitário Darcy Ribeiro Brasília-DF, Brasília, 70910900 DF Brazil

**Keywords:** Social reintegration, Women, Transgender women, Prisons, Brazil, Qualitative research

## Abstract

**Background:**

Social reintegration relies on the support given to prisoners not only during their reentry into society but also throughout their imprisonment. Our goal was to analyze the expectations reported by cisgender and transgender women returning to society and of the justice and social welfare professionals from the Brazilian prison system.

**Methods:**

A qualitative analysis using saturation sampling was conducted. The participants were selected through a non-probabilistic sampling technique. Data was collected through semi-structured interviews with professionals involved in the management of the prison system and female former inmates. Interviews were transcribed and analyzed using an open and focused coding process. Textual data was stored, organized, and coded using Atlas software according to emerging themes.

**Results:**

The study involved 15 professionals and 13 female former inmates, five of them identified as transgender women. Among the professionals, the age range went from 38 to 65 years old; they reported a work history in their respective fields, from 10 to 35 years, with an equal distribution across genders. As for the female former inmates, their ages ranged from 24 to 42 years old, and the most reported crime was drug trafficking. Their incarceration time varied from 1 to 8 years. Female inmates were vulnerable to abuse and violence, including physical, sexual, and emotional violence. Women in situations of prior vulnerability faced additional challenges during their sentences. Transgender women were even more neglected and discriminated against by the system. Despite the professionals being aware and concerned about vulnerabilities and the need to improve the reintegration process, in general, they were not sensitive to the gender perspective. There were no specific policies able to support social integration for this public.

**Conclusions:**

Data showed multifaceted challenges faced by female former inmates within the Brazilian prison system, highlighting the insufficient policies for both cisgender and transgender women. Additionally, the results revealed a lack of sensitivity among professionals regarding gender issues and their particularities in the prison system and social reintegration. These findings emphasize the need for a more comprehensive and intersectional approach that addresses the diverse socio-economic backgrounds of these individuals.

## Background

Globally, the estimated number of incarcerated individuals surpasses 11 million, with the United States, China, Brazil, Russia, and India leading in absolute prisoner counts. Women account for an average of 6.9% of this global incarcerated population. However, the number of women is rising more rapidly than the number of men in many regions (Fair & Walmsley, 2022). Regarding transgender individuals, statistics are less clear due to the lack of systematic data collection, but studies indicate that they face high incarceration rates and violence within the prison system in many countries (Hochdorn et al., 2018).

In Brazil, the prison system is divided into the federal penitentiary system, managed by the Ministry of Justice and Public Security, through the National Penitentiary Department, and the State and Federal District Penitentiary Systems, managed by the Executive Branch of the states and the Federal District (Achutti, 2017). Assessing the effectiveness of the rehabilitation system for male and female prisoners in Brazil can be complex. While there have been efforts to improve rehabilitation programs and initiatives within the prison system, challenges such as overcrowding, inadequate resources, physical and psychological violence often hinder their effectiveness (Andrade et al., 2015). In 2023, there were 27,608 incarcerated women in Brazil, of whom 53.1% were mothers. Women typically represent an average of 3–7% of the incarcerated population. Most incarcerated women are Black, have low levels of education, and 3 out of every 10 have not even received a trial, referred to as pretrial detainees (SISDEPEN, 2023). It is worth noting that, in recent years, the number of incarcerated women in Brazil has grown at a rate much higher than that of men, following the international trend. Concerning transgender women, the lack of official data makes it challenging to determine the exact number within the Brazilian prison system. The marginalization and criminalization they face in society might result in a higher likelihood of incarceration (Hochdorn et al., 2018; Coppola, 2023; Daken et al., 2023).

Prison conditions worldwide are harsh, with little improvement. Instead, there is a trend towards a punitive focus, leading to increased overcrowding, degraded facilities, and worsened quality of life (Wacquant, 2009; Quintanilha & Villen, 2019). In Brazil, hyper-detention is an instrument of social control; in a country with one of the worst inequality indexes in the world, the system that structures mass imprisonment is the most fertile ground for human rights violation practices (United Nations, 2014; Quintanilha & Villen, 2019).

The successful reintegration of prisoners into society relies on upholding their human rights as they transition from prison to community and prioritizing the individual’s rehabilitation over the crime they committed (United Nations, 2014; WHO, 2020). Although male and female former inmates struggle with reintegration, women face unique and often more complex difficulties. Heightened societal discrimination and stigma, rooted in gender stereotypes, often result in family rejection, social banishment, and, in certain jurisdictions, loss of parental rights (Flores & Pellico, 2011; United Nations, 2014; Cúnico & Lermen, 2020). Furthermore, many women’s prisons are adaptations of facilities originally built for men, resulting in inadequate conditions to meet the specific needs of women, such as medical care, privacy, and hygiene (Colares & Chies, 2010).

Gender-related issues within the prison system continue to be challenging, and the situation aggravates when it is included in the equation of transgender women within the prison system. In addition to the adversities faced by cisgender women, transgender women encounter additional challenges due to the lack of understanding and respect for their gender identities (Cúnico & Lermen, 2020; Donohue et al., 2021; Van Hout, 2022). In Brazilian prisons, transgender women often fall victim to violence, sexual abuse, discriminatory treatment, and a lack of access to adequate healthcare services. These women face a dual marginalization, both as women and as transgender, which results in increased vulnerability and difficulty in social reintegration after the period in prison (Donohue et al., 2021; Zucchi et al., 2019).

In 2020, the first penitentiary survey focused on the LGBTI + community was published, revealing that out of 1,499 establishments consulted, only 106 units (all male) reported having specific spaces for the custody of cisgender homosexual men, bisexuals, transvestites, trans women, and transgender men including a population of LGBTI population of 12,356 individuals (Brasil, 2020). In 2019, the Supreme Federal Court ordered the transfer of trans women to female prisons or reserved areas in male prisons to ensure their safety (Supreme Federal Court, 2019; CNJ, 2021). However, the implementation of these guidelines is still flawed, revealing challenges in the implementation of public policies that ensure the dignity and protection of the human rights of this vulnerable population (CNJ, 2020; Carvalho et al., 2020; Correia, 2021).

By recognizing the intersectionality of gender, gender identity, and criminal justice issues, it is possible to work towards a more equitable prison system and ensure that all prisoners, regardless of their gender identity, have equal opportunities for reintegration and rehabilitation in society (Flores & Pellico, 2011; United Nations, 2014; Cúnico & Lermen, 2020; Van Hout, 2022).

Constructing gender-sensitive risk assessments in prisons requires understanding the unique dynamics of female criminality, which differs from male patterns. Women’s criminal behavior is often contextual, rather than purely quantitative, influenced by social, economic, and psychological factors. Recognizing these differences is vital for effective rehabilitation strategies. The concept of intersectionality, which examines how various social identities intersect to create systems of privilege and oppression, is crucial for understanding the complex social injustices faced by incarcerated individuals, especially in regard to class, race and gender categories (Crenshaw, 1989; Davis, 2008; Cho et al., 2013). Integration of this framework into the prison system is essential, particularly for vulnerable women who often have low education, limited resources, and are predominantly single mothers, with a disproportionate representation of Black or mixed-race individuals (Diuana et al., 2017). Moreover, gender as a perspective that encompasses both biological and social dimensions must be thoroughly discussed in prisons. Including trans women in this study aims to present their perspectives, as they face even greater stigma and prejudice in society. Additionally, they have the same right to be recognized as women as cis women do and these layers of discrimination necessitate a nuanced approach to rehabilitation that considers the intersection of gender, race, socioeconomic status, and other identity factors (Summersell, 2018). Such an approach ensures that the distinct needs of both cisgender and transgender women are addressed, promoting more equitable and effective rehabilitation outcomes.

However, there is limited information available regarding the process of social reintegration from the perspective of former inmates themselves, concerning their experiences and needs regarding available programs. As they are a minority in Brazilian prisons, the situation is still more invisible for cisgender and transgender women. Similarly, the gender perception of professionals working within the prison system is also underexplored. In this context, this study aims to analyze the perspectives reported by cisgender and transgender women when returning to society and by justice and social welfare professionals working in the prison system.

## Methods

### Research design

This study presents a qualitative analysis using saturation sampling, identifying potential interviewees based on their direct or indirect involvement with the prison system and with cisgender and transgender women former inmates. The study intends to systematize the contexts of social reintegration for these women who have been incarcerated. Interviews were conducted with justice and social welfare professionals involved in the prison system in Brazil and with cisgender and transgender women former inmates. These interviews sought to systematize, from both the professionals’ and the former inmates’ perspectives, the practical actions regarding social reintegration and their journeys and experiences in this process.

### Participants and data collection

Participants were selected based on their availability and voluntary willingness to take part in the study. To invite participants, a network of contacts composed of individuals familiar with the criminal justice sector was invited to help identify justice and social welfare professionals in the chosen locations. The female former inmates were identified and chosen through a network of contacts within the prison system and non-governmental organizations working with this population. The interviewees were from different Brazilian cities, including Fortaleza, Brasília, Vitória, Rio de Janeiro, and Rio Grande do Sul. All cities included at least one professional and one former inmate.

Initially, the project was presented to the network of contacts to facilitate their understanding of the process and awareness of its importance (Silva et al., 2006). Subsequently, approaches were made to explain the project to the professionals and to selected female former inmates invited to participate in the study. The initial approaches and interviews were conducted virtually or in private locations chosen by the interviewees, such as their workplaces or other places where they felt more comfortable answering the questions. In general, the interviews lasted around 45 min, though they were shorter when conducted with professionals. Precautions were taken to ensure the confidentiality and privacy of research participants.

The participants’ sample was selected through the non-probabilistic sampling technique known as “snowball sampling.” Data was collected through semi-structured individual interviews. The interviews aimed to capture the discourse of professionals involved in penal execution policies to understand, from the perspective of these actors, how reintegration programs have been developed within penal execution policy. They were based on a document on social reintegration measures prepared by the United Nations Office on Drugs and Crime (United Nations, 2006). The themes proposed to the professionals were (1) What measures are available to guarantee the social reintegration of people deprived of their liberty in Brazil? Is there any difference between these measures in relation to the graduate’s gender? (2) In your opinion, what measures should be implemented to improve women’s access to social reintegration? How would you describe the level of coordination between the prison system and other governmental and non-governmental sectors in relation to these social reintegration measures? (3) What would be the main challenges to improving social reintegration and reducing the recidivism of women in the prison system? Would you like to mention any successful experience of women’s social reintegration? Questions proposed to the former inmates were different and tried to approach their personal experiences: (1) Could you tell me about your life after leaving the prison system? What were the difficulties you faced in your family context, in your relationship with your children, and with your community upon your return? (2) In your opinion, what were the main challenges for your social reintegration? Could you tell me about any positive experiences you have had in this process of returning to society? (3) Did you receive any support from the system or any other source (non-governmental organization) to return to society? What measures were offered to you to guarantee your return to society? (4) In your opinion, what other measures should be implemented to improve the process of returning to society?

### Data management plan and analysis

The interviews were transcribed and analyzed using an open, focused coding process (Nico et al., 2007; Chun Tie et al., 2019). In vivo codes, which use the respondents’ own words, were assigned to expressions through a direct analysis of the content extracted from the interviews. Based on the in vivo codes, focused code categories that exemplify specific themes were identified, as well as specific models appropriate for use in interventions. Textual data was stored, organized, and coded according to emerging themes from the qualitative data analysis using Atlas: ti software, Version 23.2. The understanding and interpretation of coded texts followed the next steps, succeeding the hermeneutic-dialectical principles outlined by Minayo (2008): intensive reading and rereading of texts to become familiar with their content and overall view; classification of accounts, cutting and pasting of text as per the relevant thematic nuclei identified, creating analysis categories following the theoretical framework of the study and its objectives; identification of meanings attributed by participants to the raised issues, seeking to understand the internal logic of this group; comparative dialogue with the literature; final interpretation with historical, social, and spatial contextualization.

### Ethical considerations

All professionals and female ex-offenders from the prison system who were contacted were invited to participate voluntarily in the study, and those who accepted signed a consent form. This project followed the ethical standards established in Resolution 466/2012 of the National Health Council, and it was submitted and approved by the Research Ethics Committee of the University of Brasília under protocol number 5.293.302/2022.

### Profile of the study population

Regarding the female former inmates in the study, they ranged in age from 24 to 42 years, and most of them were black and brown. Of the 13 female former inmates, five of them identified as transgender women. The most reported crime among this group was drug trafficking. Their periods of incarceration varied from 1 to 8 years (as detailed in Table 1).

Among the criminal justice and social welfare professionals, the age ranges from 38 to 65 years old. These professionals reported an extensive work history in their respective fields, ranging from 10 to 35 years old. There was an equal distribution across genders, but most of them defined themselves as white color (Table 2).

**Table 1:** Characteristics of the interviewed female former inmates in the study (*n* = 13)

Former inmates					
Identification	Age (Years)	Type of Crime	Race	# times in prison	Total time in prison
#1	24	Drug trafficking	Black	01	2 years
#2	27	Drug trafficking	Brown	02	4 years
#3	26	Theft	White	05	3 years
#4	36	Embezzlement	Black	02	3 years
#5	34	Drug trafficking	Black	01	2 years
#6	42	Drug trafficking	Black	01	8 years
#7	32	Drug trafficking	Brown	01	3 years
#8	40	Drug trafficking and Theft	Brown	03	5 years
#9*	26	Drug trafficking	Black	01	5 years
#10*	34	kidnapping	Black	03	8 years
#11*	24	Theft	White	02	3 years
#12*	32	Embezzlement	Black	02	2 years
#13*	33	Drug trafficking	Brown	01	1 year

*Trans woman

**Table 2:** Characteristics of the criminal justice and social welfare professionals interviewed in the study (*n* = 15)

Justice professionals with interface in the prison system					
Identification	Age (Years)	Sex/Gender	Race	Profession	Time of work in the position
#1	53	Female	Brown	Director at the penitentiary system	15 years
#2	64	Male	White	Police officer	30 years
#3	42	Male	White	State prosecutor	12 years
#4	46	Male	White	Federal judge at the Criminal Court	19 years
#5	38	Male	White	Lawyer at the Criminal Execution Court	11 years
#6	51	Male	White	Federal Prosecutor	26 years
#7	42	Female	White	Correctional officer	15 years
#8	58	Female	White	Psychologist at prison’s system	21 years
#9	65	Female	White	State prosecutor	35 years
#10	59	Female	White	Court-appointed lawyer	30 years
#11	52	Male	White	Police officer	22 years
#12	43	Female	White	State judge at the Criminal Execution Court	10 years
#13	56	Female	White	State prosecutor	25 years
#14	54	Male	Brown	Court-appointed lawyer	23 years
#15	38	Female	White	State judge at the Criminal Execution Court	10 years

## Findings and discussion

The results of this study showed the vulnerability of female inmates in the Brazilian prison system. They were mostly black, usually came from low-income, had low educational levels, had early experiences of sexual and physical violence, and did not have a network of support. These experiences are linked to mental health issues, substance abuse, and post-traumatic stress disorder (Green et al., 2016). Results also showed that there is no gender-specific reintegration policies nor professionals’ perspectives of the problem, only initiatives at state levels, even though men and women may face different challenges when seeking reintegration after serving a sentence (CNJ, 2021).

### Recovering the voices of the protagonists: former inmates from the prison system

The experiences shared by former female inmates shed light on the multifaceted challenges they face during and after incarceration. Social reintegration often proves to be an uphill battle, with significant challenges in finding employment, rebuilding family and community ties, and overcoming societal stigma. The narratives underscore the critical need for support systems to aid in this transition. Despite the difficulties, there were instances of positive experiences, notably when family, friends, or support groups provided essential encouragement and assistance. The accounts also reveal the pervasive negative perception and lack of support from the prison system and broader society, highlighting the urgent need for comprehensive measures to facilitate successful reintegration, focusing on education, employment opportunities, and emotional support. The emphasis on dignity, access to education, and skill-building resonates as fundamental pillars in empowering former inmates to navigate life beyond incarceration. Moreover, the narratives stress the vulnerability of women prisoners, particularly those who have suffered prior abuse, exploitation, or trafficking. The complexities faced by transgender women further underscore the need for tailored support and recognition within the justice system.

In their work about the Power of Violence in War and Peace, Scheper-Hughes and Bourgois (2004) explores the normalization of violence in marginalized communities and how this violence manifests in individuals’ daily lives. Bourgois argues that structural and symbolic violence perpetuates oppression and social exclusion, creating a cycle of poverty and marginalization. He discusses how these forms of violence are deeply rooted in social and economic inequalities, influencing individuals’ perceptions of their worth and potential for change. This perspective is essential for understanding the difficulties faced by incarcerated women in Brazil, who are often victims of systemic violence and find few opportunities for social reintegration after serving their sentences. Furthermore, they highlight the importance of recognizing the different forms of violence that affect marginalized communities, including everyday violence, symbolic violence, and structural violence. He emphasizes that these forms of violence are interconnected and reinforced by institutional policies and practices that neglect the needs and rights of marginalized individuals. In the context of the Brazilian prison system, this analysis helps to understand how violence is normalized within prisons and how social reintegration policies need to address not only the immediate needs of former inmates but also the structural factors that perpetuate their marginalization. Integrating these perspectives into policy development can help create more effective and just solutions for the reintegration of these women into society.

In total institutions, such as prisons, there is a fusion of the different spheres of a person’s life, where symbolic worlds are reduced to one (Goffman, 1961). This is significant because the environment of these institutions determines a particular logic that defines the interactions of those who reside in them, granting specific identities to individuals (beyond their own, such as gender), such as converting, for the case study, a citizen into a prison inmate. These accounts underscore the pressing need for systemic change, emphasizing the importance of a holistic approach to support the social reintegration of women post-incarceration, addressing their unique challenges, and fostering a more inclusive environment.

### Experiences after imprisonment

Former inmates answered questions about their experiences after leaving the penitentiary system.

*“My life got even harder. I could not find a job; my friends and family ditched me after I got locked up. Out on the street, clients don’t want to hook up with me anymore just because I’ve been in prison. How am I supposed to pay my bills? It is really tough! Sometimes, you feel so trapped that the thought of going back to the hustle crosses your mind”. (Former inmate #2).*

*“The label “ex-con” ruins everything, you know? It messes up any chance of getting a job and even finding a boyfriend, believe it or not (laughs). And let me tell you, having a job is the bare minimum to keep moving forward – especially when you lose your family’s and friends’ support. We need a job to stand on our own two feet. I used to have a job, even though it sucked. Now, I’ve got neither a job nor family nor friends, just left with sadness and regret – I feel so small”. (Former inmate #11)*

Female prisoners face a range of specific issues that impact their experience and their reintegration into society. These issues encompass detention conditions, gender-related demands, and the effects on their mental and emotional well-being (Bartlett & Hollins, 2018). When it comes to the situation of transgender women; the access to health is even worse, causing them to be excluded from the system (Winter et al., 2016). Social vulnerability encompasses a state of fragility and dependence arising primarily from adverse socio-economic conditions that certain groups endure. A particularly affected group within this framework is women, especially those with past affiliations with the prison system. Many of these women, who often serve as mothers and occasionally as single mothers and primary household providers, fight not only with challenges linked to their gender identity but also with societal prejudice. Judith Butler’s exploration of “precarity” and Martha Albertson Fineman’s “Vulnerability Theory” offer insightful examinations into the societal positioning of marginalized groups. Butler’s concept sheds light on the societal constructs that regard certain lives as less valuable, a harsh reality experienced by many incarcerated women from underprivileged backgrounds (Butler, 2016; Fineman, 2013).

### Family and community support in the reintegration process

Some women found support from fellow inmates or their own family and friends, facilitating the transition process. However, when the female former inmates were asked about the challenges they faced in the family context, in their relationships with their children, and with their community upon their return, the responses were mainly focused on negative experiences, which were even deeper for transgender women. Based on observations from this study, it appears that women in prison often receive fewer visits compared to their male counterparts, as they are typically the ones who visit their partners when roles are reversed. Consequently, the conviction and time spent in prison can strain family relationships, resulting in tensions and difficulties in reestablishing healthy family bonds. Moreover, the social stigma associated with criminal involvement can lead to issues of acceptance and exclusion from the community.

*“They do not accept me, and they do not even want me in their lives. They do not know or care that there are days when I have nowhere to sleep and no food. It is funny, you see? Because I used to visit my brother in jail, before I got in, and once I was the one locked up, I had nobody visiting or caring for me!” (Former Inmate #12)*.

*“I have not been in touch with them for a long time! Since I left home to try my luck in the city, I lost contact with them. They never cared about me. It was such a tough life for all of us, especially after we lost my father. I had a child when I was young, but he died of pneumonia or something like that. I am all alone in this world. My friends are the ones I made in prison and later in the support group. We are together, trying to turn our lives around and get a fresh start”. (Former Inmate #3)*

*”The difficulties are many. My family does not accept me; they reject me and constantly tell me to go back to prison, saying that it is where I belong. They believe that prison is waiting for me again. There are times when I think I’m going crazy “. (Former Inmate #7)*

*”What family? What friends? What community? Nobody helps you when you are at your worst! I suffered violence from my family and my partner. I feel very alone and sometimes I cry”. (Former Inmate #13)*

While it is common for female former inmates to face family and community issues during the process of social reintegration, as their conviction and time spent in prison can strain family relationships and make it challenging to reestablish healthy family bonds, along with the social stigma associated with criminal involvement leading to acceptance issues and exclusion from the community.The stigma (Goffman, 2003) associated with being an ex-offender will accompany the inmates when they leave prison and will have inevitable consequences in the formation of their new identity. By conditioning interactions with people outside the prison, the stigma becomes internalized, in the manner of a self-fulfilling prophecy, and shapes a new identity that may or may not have a propensity to continue committing crimes. The implicit criticism of the resocializing function of prison seems evident.

Nevertheless, in the interviews, there were positive responses that highlighted family and societal support as crucial elements for the social reintegration of these women.

*“My family was very supportive of me. When I was in prison, my biggest issue was that, at the time, I had a two-month-old baby, and being away from her was tough. She gave me the strength not to make the same mistakes again”. (Former Inmate #8)*

*”I did not have any problems with family and friends, no! But I know it is common for many women to face issues with their families and neighbors when they get out of jail. I had some friends in that situation. Besides, there is much prejudice out there.“. (Former Inmate #4)*

*”Help from the system? In jail? (laughs) I took a sewing class, but I was not very good at it (laughs). What helped me was the support group for people getting out of prison. They were essential to me. I always talk about them to other friends who are having problems. There are some nice people there. They help many people turn their lives around. They give us a chance”. (Former Inmate #1)*

The family support and the shared institutional experiences laid the foundation for forming strong bonds, exemplified by some interviews. The relationships, formed amidst the unique challenges and shared experiences of institutional life, became positive anchors, offering support and avenues for transformation post-release. Social support not only helps reduce recidivism but also plays a fundamental role in former inmates’ emotional and psychological well-being (Freudenberg et al., 2008; Cúnico & Lermen, 2020). In “Asylums,” Erving Goffman delves into the dynamics of relationships within “total institutions.” These environments induce a process termed “mortification of self” — systematically breaking down an individual’s identity, subsequently replaced by one crafted by the institution. In such settings, the shared experiences and the unique culture that develops catalyze deep and enduring connections among members (Goffman, 1961).

### Challenges for social reintegration

When the discussion focused on the main challenges they faced for social reintegration, and they were asked to share their positive experiences returning to society, abandonment was a common theme. Any support, when available, was greatly appreciated.

*“Everything is a challenge! No one accepts you, wants to be your friend, or gives you a job. It is terrible! In adult school, it is hard to find a spot, and they look at you suspiciously if they know you have been in prison. We are marked!“. (Former Inmate #7)*

*”One of the biggest challenges was overcoming the prejudice because of my conviction. Many people had prejudice and fear of me and did not want to give me a second chance. It made it difficult to find a job and start a new life. Even at the health clinic, they treated me differently. It is like we’re contagious”. (Former Inmate #10)*

*”The good experience in all of this was finding the support group that encouraged me and gave me the chance to change my life, and even managed to find me a job! They cared about me, and that was great. They even got me a doctor for an exam; I had never had that chance before. I thank God every day because a friend - now locked up again -gave me their contact”. (Former Inmate #4)*

*”I don’t know how it is nowadays, but in my time in prison, there was no support, or if there was, I never heard of it (laughs)! I was so depressed in prison that I did not think of anything other than getting out of there as soon as possible. But I do not remember anyone asking if I needed any help”. (Former Inmate #8)*

The weight of being labelled a “former inmate” is keenly felt among these women. Criminal labelling theory suggests that these stigmatizing tags, once internalized, can profoundly alter one’s self-perception, influencing the trajectory of societal interactions and opportunities (Zucchi et al., 2019). Some data from the interviews underscore the manifold challenges that those bearing the “former inmate” label encounter. They face entrenched societal biases and find barriers in spaces that ideally should offer healing and growth, such as health clinics or educational settings. The deprivation of essential societal anchors like education, health, jobs, and relationships accentuates their emotional and practical burdens. To truly foster reintegration, society must look beyond these reductive labels, acknowledging the richness of individual experiences and the vast potential for positive change (Hochdorn et al., 2018; Zucchi et al., 2019). Paul Farmer’s concept of “structural violence” underlines that some groups are made vulnerable by the historical and societal dynamics that frame their existence (Farmer, 2005). This vulnerability stands starkly apparent for women reemerging into society after serving time in the Brazilian prison system.

The situation becomes more aggravated for transgender women. Already marginalized due to their gender identity, they encounter magnified stigmatization upon release. Such multifaceted marginalization is not merely a result of individual prejudices but exemplifies a deeply rooted structural violence. The symbolic interactionism lens offers deeper insights: it is not just about the societal structures but how interactions within these structures and the meanings they uphold fuel the structural violence that released women, especially transgender women (Cúnico & Lermen, 2020; Snacken et al., 2022).

### Support for social reintegration received from the prison system or other institution

When asked about the support from the prison system or some other source (non-governmental organizations or international agencies) for reintegration into society and what measures were offered to ensure their return to society, opinions were mostly negative. However, a few of them had access to support, which demonstrates that despite the existence of measures, they were insufficient and incapable to reach all the former inmates.

*“No, who cares about us? They don’t even know we exist. It’s really tough to feel invisible and unimportant to everyone”. (Former Inmate #5)*

*“I haven’t seen much positive at all. I’m trying to learn how to deal with the freedom of going where I want without asking for permission and without being mistreated. I was seen like an animal, and now I’m trying to learn how to be human again”. (Former Inmate #3)*

*“Are you kidding me? I’ve only received judgment and contempt. Nobody likes an ex-con! Life is already hard when you’re poor and uneducated, but when you’ve been to prison, things get even worse. If you’re a trans woman, forget about it. Who wants a former inmate around? Everyone’s afraid we’ll steal or do something wrong against them”. (Former Inmate #9)*

*“When I got out of prison, it was night, and they didn’t even give me a bus pass or offer a ride. I’m looking for a positive experience to hold onto and move forward! There’s nothing good! People are afraid of ex-convicts, and the system doesn’t care about us”. (Former Inmate #10)*

*Having support was crucial. My family and friends encouraged me and believed in my ability to change. I also participated in social reintegration programs that provided career guidance, training, and emotional support. These resources helped me develop skills, build my confidence, and have access to job opportunities”. (Former Inmate #6)*

*“No, not even a grain of rice. Prisoners are worthless and have no use, former prisoners either. We only cause trouble. The government wants to get rid of us, and people on the outside want us to stay there so we won’t bother anyone. (Former Inmate #13)*

These women, even before being incarcerated, already faced prejudice related to their social conditions. The fact that they now reintegrate into society with the label of “former inmate” only exacerbates the existing prejudices. In this sense, the intersectionality theory delves into how intersecting social categories, notably race, gender, and other individual identities, overlap and produce systems of inequality and discrimination. Within the context of these formerly incarcerated women, multiple forms of oppression converge (Crenshaw, 1989). Former inmate #9, a transgender woman, mentioned how her gender identity already marginalizes her, with this marginalization further amplified by her status of a former inmate. Racially speaking, there is a marked disproportionality in many prison systems, which, when coupled with other identities such as gender or disability, layers on additional oppression. Thus, the experience of a released woman is multifaceted and shaped by multiple identities at once, epitomizing the crux of intersectionality (Flores & Pellico, 2011).

The perception of the importance of measures to enhance the reintegration process into society was largely associated with education and employment. Health was scarcely mentioned, suggesting that basic needs for sustenance take precedence, with concerns about access to healthcare arising subsequently.

*“Helping us find a job to start over. We need dignity, the chance to study, to learn how to read and do some basic math. I never had any of that, not even before I got locked up, and life only gets tougher, you know?”. (Former Inmate #11)*

*”I think it’s essential to provide more opportunities not to end up back in jail. We need a chance to learn something, take some course, learn a trade, and get some support. We have to learn to stay out of trouble (laughs)”. (Former Inmate #2)*

*”Oh boy, there’s a lot to be done still! The courses they offer are good, but they don’t always help! My life was already hard even before I got locked up, and it didn’t get any better afterward. There needs to be more preparation to get us ready for life on the outside, we need to be aware that we’ll face discrimination. School is also good for us to make us smarter and help us defend ourselves in the world, and maybe find a job eventually”. (Former Inmate #5)*

*“I’d say it’s essential to believe in ourselves and that we can change our lives, even when nobody else believes in us. But having support from groups or organizations that work with people getting out of prison is crucial because they help us organize our lives, find jobs, school, and medical care. We also need to be willing to change and be patient because it can take time to rebuild our lives. But we can’t let our past define our future”. (Former Inmate #12)*

In this process, it was observed that women prisoners often find themselves in situations of vulnerability related to the lack of adequate protection and inhumane treatment they suffer. In the case of women who have previously suffered different types of violence, such as victims of abuse, human trafficking or sexual exploitation, they may face additional challenges during their sentences. When addressing the issue of transgender women in this process, the situation can be even more complex, as they are further invisibilized and discriminated against by the system.

### Perspectives of criminal justice and social welfare professionals

This section describes the perspectives of criminal justice and social welfare professionals regarding their inputs and points of view. Their perspectives highlight the importance of measures for improving female inmates’ social reintegration, challenges related to social inequalities, coordination of the prison system with other sectors, and reintegration measures to prevent recidivism. Efforts include involvement in income-generating projects, emphasis on education and healthcare, concerns about gender-based differences, varied coordination levels, and initiatives to reduce recidivism through education, mental health support, and family ties. Successful experiences such as the “Art in Prison” Project and the “Começar de Novo” initiative aim to facilitate job market reintegration and promote social inclusion for former inmates.

### Gender in prison perspectives

Women in the prison system often face gender inequalities. The lack of specific programs to address their needs, such as adequate healthcare, maternity support, family planning, and access to reproductive health services, contributes to the perpetuation of these inequalities. When asked about the existence of gender-based differences in these measures for former inmates, professionals responded:

*“The law does not make a distinction between genders but, in practice, each state in the federation can implement specific programs for each gender. This decision is up to regional and local authorities. But no, at the federal level, there is no separation by gender. Everyone has the same rights and responsibilities” (Professional #2).*

*“I do not think so. To be honest, I do not even think it is necessary. Everyone needs employment, education, healthcare, and access to citizenship. Why would it be different by gender? The problems they face are the same! To be completely honest, our measures do not work well in general, and thinking about gender differences now would distract even more the focus from what needs to be done. Do not get me wrong, but it is better to focus on the general population than to try to improve particular conditions. If you want to change the situation, you need to address where the problem is more significant, in this case, among men, who make up most of the inmates in the prison system”. (Professional #13)*

*”By law, no. However, at the state level, there are programs that take these differences into account. Honestly, I believe that as important as they are, we need to be vigilant not to create even more inequalities”. (Professional #10)*

In this study, little concern for gender differences in prisons was observed among the interviewed justice and welfare professionals. Lacking interest in gender differences in prisons by justice professionals is a multifaceted problem, rooted in a lack of training for awareness, biases, limited resources, and a resistant institutional culture. If gender issues were not a concern for the professionals, even less attention was given to differences concerning trans women, as this topic was not mentioned by any of the professionals during the interviews. This lack of concern can negatively impact both the incarcerated individuals and the overall effectiveness of the penal justice system, affecting rehabilitation (Belknap, 2014; Quiroga-Carrillo et al., 2024). Gender is recognized as a fundamental social determinant of health, significantly influencing health policies and the delivery of equitable healthcare for all. Attention to the concepts of ‘sex’ and ‘gender’ is increasingly seen as essential for advancing scientific understanding of health inequities and outcomes. The integration of these considerations into health research not only strengthens the overall health evidence base but also facilitates the development of specific health policies and planning (Gahagan et al., 2015).

### Social reintegration policies

When asked about the availability of social reintegration policies for inmates in Brazil, a police officer with ten years of experience in the field responded:

*“Well, in that regard, we have the Penal Execution Law, Law 7,210, of July 11, 1984, which establishes the means to achieve the social reintegration of inmates, aiming to prevent crime and guide their return to society, ensuring material, legal, educational, and other forms of assistance. Implementing these measures that contribute to the reintegration of the individual into social life has a significant role for the person who committed the offence and is also extremely important for society. It significantly reduces the likelihood of recidivism; after all, this individual is someone’s neighbor; their criminal actions can potentially affect any of us”. (Professional #3)*

A prison director for over 20 years believed that the process of social reintegration should start during the incarceration period, and opportunities should be offered equally to ensure an effective process.

*“The process of social reintegration should be facilitated at the moment the person is deprived of liberty. With different strategies, some prison units allow inmates to participate in income-generating projects. The issue is that this does not happen uniformly within the system. Some units have installed factories, gardens, craft workshops, baking facilities, automotive workshops, computer labs, and so on. Even though some of them may not work – There is a beautiful bakery inside the penitentiary in Brasilia that is simply closed. Generally, an internal selection process is carried out and, those who manage to participate, receive compensation and may also benefit from sentence reduction” (Professional #1)*.

It was observed that the interviewed professionals understood that the social reintegration of former inmates was, if not the sole purpose of sentence, one of its most important objectives, or at the very least, the most appropriate one. However, the gender perspective did not emerge in their point of view. Some responses might even divert attention from the strategies that need to be implemented. In their review, Cúnico and Lermen (2020) revealed a disparity in the application of gender theory in the context of prisons. While academic progress has been made in the theoretical discussion of gender as a social construct, this progress was not consistently reflected in prison-relatedresearch.

### Coordination of the prison system and other sectors to work on social reintegration

When questioned about the level of coordination of the prison system with other government and non-governmental sectors regarding these social reintegration measures, the professionals gave the following insights:

*” At the federal level, it seems that the National Penitentiary Department has good coordination with different sectors regarding health, education, human rights, and social assistance. However, at the state level, it is very diverse. In fact, coordination among different policies would be the way to implement the National Policy for the Care of Individuals Released from the Prison System, but in practice, this is not reflected in the states. There are successful experiences, but they do not define a standard of action when it comes to the essential coordination for the reintegration of prisoners”. (Professional #12)*

*”In my work field, the justice is proactive in guiding this coordination. I cannot say it always works, but the important thing is that we always try. The system has its rules; we have the Penal Execution Law that guides our actions, and various partners have supported the cause and are concerned about it. However, I believe that leadership should always come from the judiciary, as we have the training in the field and know how to deal with these issues. The state government has been dedicated to integrating the inmates into the job market, and we have the support of the private sector. Today, in my state, we have around 200 companies that provide job opportunities for nearly 3,000 inmates, both inside and outside the prisons. This is an opportunity for the inmate to reintegrate and regain their dignity. It is also very beneficial for the entrepreneur, who receives various benefits from the state”. (Professional #15)*

*” Despite the law establishing various measures for the reintegration of inmates, the reality is that the Brazilian penitentiary system is chaotic, and very little has been done to improve women’s access to social reintegration. The State should be more involved in monitoring the reintegration process, ensuring employment, education, healthcare, and other basic rights. I emphasize that these actions fall under the jurisdiction of each state of the federation, which is responsible for organizing their care network”. (Professional #8)*

Policies and programs implementation should be focused to assist individuals in prisons in returning to their communities, preventing the commission of new crimes, and consequently, new incarceration, as well as reducing situations of vulnerability, such as illicit drug abuse, and helping them become dignified and productive members of their communities (Freudenberg et al., 2008; Snacken et al., 2022).

There are many gaps in the prison system that need to be filled. The involvement of justice professionals in this process is also important:

*“It would be important to put into practice the minimum standards established by the United Nations for the treatment of prisoners. Standardizing the care model for incarcerated individuals would be crucial. There is a framework of legislation that establishes guidelines for social reintegration in Brazil. However, the prison system in the country is quite diverse. The physical structure of the units, the way each state administers its prison facilities often does not allow for an effective social reintegration process. The system’s primary focus is always on security, and there is little flexibility on the part of authorities to balance security with the other needs of prisoners” (Professional #11)*.

*“I facilitate the reintegration of prisoners with society through community service, promoting integration, awareness, and socialization of all parties involved. I serve as a bridge between companies and industries that open their doors to receive inmates and former inmates for work activities. This is important work for the social reintegration of these individuals, but it is not easy! It is not a straightforward task because we must consider these individuals in a broader context and not focus on the crimes they committed. Trying to understand that they come from underprivileged social backgrounds and often had no better choices. In this sense, we need to convince business owners to give these individuals an opportunity to work with them”. (Professional #7)*

A federal prosecutor, who was interviewed, emphasized that the challenge of social reintegration is intrinsically linked to the social inequalities that affect individuals even before they are in prison:

*“I believe that the effective role of the State in promoting education and healthcare is essential. I am talking about a general need. If we do not increase access to basic social conditions, we will spend our time trying to fix system failures. We must admit that this is not an easy task, as social inequality in the country is a significant problem. How can we effectively improve people’s living conditions if we do not address the root of the problem? We need to reflect on this. I believe that before we talk about reintegration, we have to talk about social integration.” (Professional #6)*.

Although the Brazilian prison system is recognized as a model with its legislation considered among the most advanced in the world, ensuring the social reintegration of prisoners, there must be more clarity between the theoretical and programmatic frameworks and social processes (CNJ, 2021). Implementing the law in the country’s reality remains problematic due to a lack of investment in maintaining prison services, resulting in overcrowded facilities, and delaying reintegration measures, especially those administered by the federative states. The challenge lies in rehabilitating and reintegrating individuals into a society that, despite robust legislation, offers limited practical resources for such reintegration (Assis, 2007; Donohue et al., 2021).

### Challenges to improve social reintegration and reduce recidivism

According to the assessment of many of the professionals interviewed, the main challenge to improve social reintegration and reduce recidivism among women in the prison system is to work on the promotion of education and professional training, which leads to an improvement in the self-esteem of these women. At the same time, it is necessary to promote comprehensive wellness actions in mental health:

*“.that can provide former female inmates with self-knowledge that allows them to control their impulses of anger and aggressiveness in adverse situations. There is also a need for initiatives that strengthen the ties of these individuals with their families and community. Without family and friends, it is much more difficult to reintegrate; everyone needs a reference to move on, right?” (Professional #5)*.

*“The main challenge to improve social reintegration and reduce recidivism, not only for women, but for inmates in general, is to ensure the full implementation of measures already established in the Penal Execution Law, with investment in prison infrastructure and putting in practice prison policies aimed at preventing criminal recidivism and guiding the return to society. We must prioritize integrating these individuals into society before discussing reintegration, as they often find themselves on the fringes of community acceptance. We need to work in partnership across sectors and rectify the deficiencies of the Brazilian State in guaranteeing these rights. We still have a long way to go!“. (Professional #10)*

*”Personally, not really. I am a bit skeptical in this regard, but I know the state government has taken some initiatives in this direction. In my opinion, one of the biggest challenges in this agenda is overcoming prejudice and providing social inclusion for inmates and former inmates, creating the possibility of reducing social inequalities and crime in the State. I know that the State Department of Justice (SEJUS) has been investing in projects to reestablish inmates’ citizenship. The main one is the program called “Social Responsibility and Resocialization,” which aims to awaken and develop the skills of inmates, offering education and vocational training and trying to place them in the job market”. (Professional #6)*

*”Art in Prison” Project - Developed in Ceará state is an excellent experience. I had the opportunity to talk to the women and see the difference it made in their lives regarding self-esteem, income generation, and prospects”. (Professional #9)*

Finally, implementing adequate reintegration measures and policies is crucial to preventing recidivism. The interviewed professionals reported the existence of some successful experiences in the social reintegration of female former inmates that they were familiar with, such as:

*The National Council of Justice has been focusing its attention on the topic. I would like to highlight, among other initiatives, the publication of “The Handbook for Incarcerated Women”, aimed at clarifying the rights and duties of prisoners with clear and direct information about constitutional guarantees and legal and administrative prerogatives. This is a tool for social reintegration made free of charge by the National Council of Justice (CNJ)”. (Professional #14)*

*”Reintegration into the job market is one of the most successful experiences of social reintegration for women because this is the biggest challenge a former prisoner faces. In prisons, we should not consider work merely as a pastime, make-believe, or something of that sort, as that would not be pedagogical. A pedagogical approach would be to focus on the concept of work that aims to establish the person’s dignity as someone capable of providing for their subsistence and that of their family. It is essential for former inmates to gain constructive experience to survive without committing further crimes. An important project in this regard was proposed by the National Council of Justice to be implemented in all states of the federation; I think the name of the project is “Começar de Novo”. Nevertheless, as I mentioned before, the states of the federation are the key players in the process”. (Professional #4)*

Justice professionals should adopt a gender-informed and sensitive approach when dealing with the incarcerated population. This requires ongoing training, the development of inclusive policies, and the implementation of rehabilitation programs tailored to the specific needs of men and women. Comprehensive reforms that address gender needs can help create a more equitable and effective justice system. This includes reviewing sentencing laws and implementing evidence-based practices. Only with a deep understanding of gender differences and a commitment to equity can the justice system function more fairly and effectively for all individuals (Ballesteros-Pena & Almeda, 2015; Cruz et al., 2023).

## Conclusions

This study provides valuable insights into the common challenges of social reintegration faced by professionals and former inmates across various Brazilian regions. Despite the potential influence of social desirability bias, which may have influenced responses, as individuals often tend to provide socially acceptable answers in face-to-face interviews, and the relatively short duration of the interviews, which are limitations of this study, the findings have the potential to contribute to the improvement of policies and practices related to social reintegration within the Brazilian prison context.

Data from this study can guide strategies for former inmates in Brazil. It is important to highlight that several initiatives have been implemented in the country to support the social reintegration of women after their release. However, these units have not been able to provide the necessary services for all women during their imprisonment (Andrade et al., 2015; Miranda et al., (2022). For offering proper social reintegration, it is necessary that we not only address the immediate challenges these women face but also the systemic and deep-rooted inequalities that persist in the penal system. Enhancing detention conditions, ensuring robust healthcare provisions, safeguarding against all forms of violence, and offering ample support for familial responsibilities are non-negotiable steps towards a more equitable system.

Our findings highlight the complex challenges faced by female former inmates, aligning with, and expanding on existing literature. A holistic approach to policy and intervention is crucial, considering the diverse social identities and experiences of these women. Bridging the gap between theoretical gender-specific issues and their practical application in prisons and reintegration programs is essential. The marginalization of the gender perspective among prison professionals underscores the urgent need for educational and training interventions to enhance gender sensitivity.

## Data Availability

There are no data and material other than the manuscript.
